# Extra Virgin Olive Oil Phenolic Extract on Human Hepatic HepG2 and Intestinal Caco-2 Cells: Assessment of the Antioxidant Activity and Intestinal Trans-Epithelial Transport

**DOI:** 10.3390/antiox10010118

**Published:** 2021-01-15

**Authors:** Martina Bartolomei, Carlotta Bollati, Maria Bellumori, Lorenzo Cecchi, Ivan Cruz-Chamorro, Guillermo Santos-Sánchez, Giulia Ranaldi, Simonetta Ferruzza, Yula Sambuy, Anna Arnoldi, Nadia Mulinacci, Carmen Lammi

**Affiliations:** 1 Department of Pharmaceutical Sciences, University of Milan, 20133 Milan, Italy; martina.bartolomei@unimi.it (M.B.); carlotta.bollati@unimi.it (C.B.); icruz-ibis@us.es (I.C.-C.); gsantos-ibis@us.es (G.S.-S.); anna.arnoldi@unimi.it (A.A.); 2 Department of Neuroscience, Psychology, Drug and Child Health, Pharmaceutical and Nutraceutical Section, University of Florence, 50019 Florence, Italy; maria.bellumori@unifi.it (M.B.); lo.cecchi@unifi.it (L.C.); nadia.mulinacci@unifi.it (N.M.); 3 Departamento de Bioquímica Médica y Biología Molecular e Inmunología, Universidad de Sevilla, 41009 Seville, Spain; 4 CREA, Food and Nutrition Research Centre, 00178 Rome, Italy; giulia.ranaldi@crea.gov.it (G.R.); simonetta.ferruzza@crea.gov.it (S.F.); yula.sambuy@crea.gov.it (Y.S.)

**Keywords:** phenolic phytocomplex, ROS, lipid peroxidation, secoiridoids, hydroxytyrosol, oleuropein aglycone, trans-epithelial transport

## Abstract

In the framework of research aimed at promoting the nutraceutical properties of the phenolic extract (BUO) obtained from an extra virgin olive oil of the Frantoio cultivar cultivated in Tuscany (Italy), with a high total phenols content, this study provides a comprehensive characterization of its antioxidant properties, both in vitro by Trolox equivalent antioxidant capacity, oxygen radical absorbance capacity, ferric reducing antioxidant power, and 2,2-diphenyl-1-picrylhydrazyl assays, and at the cellular level in human hepatic HepG2 and human intestinal Caco-2 cells. Notably, in both cell systems, after H_2_O_2_ induced oxidative stress, the BUO extract reduced reactive oxygen species, lipid peroxidation, and NO overproduction via modulation of inducible nitric oxide synthase protein levels. In parallel, the intestinal transport of the different phenolic components of the BUO phytocomplex was assayed on differentiated Caco-2 cells, a well-established model of mature enterocytes. The novelty of our study lies in having investigated the antioxidant effects of a complex pool of phenolic compounds in an extra virgin olive oil (EVOO) extract, using either in vitro assays or liver and intestinal cell models, rather than the effects of single phenols, such as hydroxytyrosol or oleuropein. Finally, the selective trans-epithelial transport of some oleuropein derivatives was observed for the first time in differentiated Caco-2 cells.

## 1. Introduction

Oxidative stress, which refers to the shift in the oxidants/antioxidants balance in favor of the formers, contributes to many pathological conditions [1]. Aerobic organisms have integrated antioxidant systems, which include enzymatic and nonenzymatic antioxidants, which are usually effective in blocking the harmful effects of reactive oxygen species (ROS). However, in pathological conditions, the antioxidant systems can be destroyed and the consequent increase of intracellular ROS levels contributes to the development and progression of many chronic and non-communicable diseases. The use of food-derived antioxidants may represent a strategy to cope with the progression of diseases related to oxidative stress [2]. In fact, experimental, clinical, and epidemiologic studies have shown that the consumption of specific food phenols is positively linked with health-promoting effects [3]. 

The consumption of extra virgin olive oil (EVOO) has been largely associated with numerous health benefits [4]. It has been frequently reported that olive oil has anti-inflammatory, neuroprotective, and immunomodulatory activities [5] and can reduce the risk of coronary heart disease by modulating the high-density lipoprotein (HDL) cholesterol levels [6]. The literature indicates that these beneficial effects are due, at least in part, to the presence of some hydrophilic components such as phenols, which are well-recognized for their remarkable antioxidant activity [7]. Among these phytochemicals, hydroxytyrosol (OH-Tyr), tyrosol (Tyr), and oleuropein (Ole) have the greatest antioxidant activity and capacity of reducing oxidative stress [8]. In particular, EVOO decreases ROS and malondialdehyde (MDA) production [9] and NO release [10] and reduces the expression and production of the inducible nitric oxide synthase (iNOS) and cyclooxygenase 2 (COX-2) [11]. The European Food Safety Authority (EFSA) has published a positive opinion on the health claim that “Olive oil polyphenols contribute to the protection of blood lipids from oxidative stress”, determining that 5 mg of OH-Tyr and its derivatives (e.g., Ole and Tyr) in olive oil should be consumed daily for a sufficient avoidance of oxidative damage [12]. 

In this context, the present investigation was conducted on a phenolic extract (BUO) obtained from an EVOO of the Frantoio cultivar cultivated in Tuscany (Italy), with a high content of total phenols. The phenol characterization, reported in a preceding paper, has been performed applying the official method of the International Olive Council [13] for quantifying total phenols content, the ^1^H-NMR analysis for evaluating the relative abundance of aldehyde derivatives of secoiridoids, and a validated hydrolytic method to evaluate the total content of OH-Tyr and Tyr, as the sum of free and bound forms [14]. These analyses have indicated that the concentration of OH-Tyr and Tyr were 208.0 ± 15.6 and 156.0 ± 3.9 µg/g of dried extract, respectively, and that the Ole derivatives were greatly prevalent (444.9 µg/g) [15]. The same study has also shown that the BUO extract modulates cholesterol metabolism in human hepatic HepG2 cells, through direct inhibition of the activity of 3-hydroxy-3-methylglutaryl-coenzyme A reductase (HMGCoAR) and consequent activation of the low-density lipoprotein receptor (LDLR) pathway [15]. To express their activities, food phytochemicals need to be bioavailable. This means that, after ingestion, they have to be absorbed by enterocytes to reach the target organs and display their biological activity. Despite their established physiological importance, the literature clearly underlines some limitations and a gap of knowledge on food phenols absorption and metabolism, especially when they are within a phytocomplex. Indeed, when they are consumed within the diet, phenols may undergo numerous structural modifications and their properties may be affected by the interactions with other constituents of the food matrix. The interactions with the digestive enzymes can alter their availability, and domestic processing appears to have important effects on the total phenol content and activity [16]. Interestingly, recent data indicate that a combination of phytochemicals, rather than any single phenolic compound, is responsible for the observed health benefits [15,17,18]. 

To promote the bioactivity of the BUO phytocomplex, the first objective of the present study was a detailed investigation of its potential antioxidant activity in vitro and on hepatic and intestinal cells, due to the physiological interplay existing between these organs. To achieve this goal, the antioxidant activity was evaluated in vitro and at the cellular level, by measuring its capacity to reduce the level of intracellular ROS, lipid peroxidation, and NO levels in human hepatic HepG2 and human intestinal Caco-2 cells, where the oxidative stress was induced by H_2_O_2_. The second objective of the study was an assessment of the intestinal transport of the different phenolic components of the BUO phytocomplex that was performed using differentiated Caco-2 cells as a model of mature enterocytes. 

## 2. Materials and Methods 

### 2.1. Materials and Cell Cultures

All chemicals and reagents were commercially available, and more details are reported in the Supplementary Materials.

### 2.2. Cell Culture

HepG2 cells and Caco-2 cells were cultured in DMEM high glucose with stable L-glutamine, supplemented with 10% FBS, 100 U/mL penicillin, 100 µg/mL streptomycin (complete growth medium) with incubation at 37 °C under 5% CO_2_ atmosphere. Caco-2 cells were routinely sub-cultured at 50% density [19]. HepG2 cells were used for no more than 20 passages after thawing, since the increase in passage number may change the cell morphology and characteristics and impair assay results.

### 2.3. Production of the EVOO Extract

An EVOO sample produced by Società Agricola Buonamici SrL (Fiesole, Florence, Italy) in the 2017 olive oil campaign from monocultivar olives of the typical Tuscan cultivar Frantoio was used for the study (BUO oil). The BUO extract was obtained following the procedures previously described [15]. See Supplementary Materials for detailed information and conditions.

### 2.4. 3-(4,5-Dimethylthiazol-2-yl)-2,5-Diphenyltetrazolium Bromide (MTT) Assay

A total of 3 × 10^4^ HepG2 cells/well and 5 × 10^4^ Caco-2 cells/well were seeded in 96-well plates and treated with 25.0, 50.0, 100.0, and 200.0 μg/mL of BUO extract, or vehicle (H_2_O) in complete growth media for 48 h at 37 °C under 5% CO_2_ atmosphere. Experiments were performed by a standard method with slight modifications [17] and more details are provided in Supplementary Materials. 

### 2.5. 2,2-Diphenyl-1-Picrylhydrazyl (DPPH) Assay

The DPPH assay to determine the antioxidant activity in vitro and in situ was performed by a standard method with some slight modifications [17]. More details are reported in Supplementary Materials. Briefly, for the in situ experiments, 3 × 10^4^ HepG2 and Caco-2 cells/well were seeded in a 96-well plate, overnight in growth medium and the following day they were treated with the BUO extract at a concentration of 25 μg/mL for 24 h at 37 °C under 5% CO_2_ atmosphere. More details are provided in Supplementary Materials where the calibration curve using Trolox has been obtained (Figure S1). 

### 2.6. TEAC Assay

The TEAC assay is based on the reduction of the 2,2-azino-bis-(3-ethylbenzothiazoline-6-sulfonic acid (ABTS) radical induced by antioxidants. The ABTS radical cation (ABTS^+●^ was prepared by mixing a 7 mM ABTS solution (Sigma-Aldrich, Milan, Italy) with 2.45 mM potassium persulfate (1:1) and stored for 16 h at room temperature and in dark. To prepare the ABTS reagent, the ABTS^+●^ was diluted in 5 mM phosphate buffer (pH 7.4) to obtain a stable absorbance of 0.700 (±0.02) at 730 nm. For the assay, 10 µL of BUO extract (at the final concentrations of 0.5, 1.0, 5.0, and 10.0 µg/mL) were added to 140 µL of diluted the ABTS^+●^. The microplate was incubated for 30 min at 30 °C and the absorbance was read at 730 nm using a Synergy™ HT-multimode microplate reader (Biotek Instruments, Winooski, VT, USA). The TEAC values were calculated using a Trolox (Sigma-Aldrich, Milan, Italy) calibration curve (60–320 µM) (Figure S1).

### 2.7. FRAP Assay

The FRAP assay evaluates the ability of a sample to reduce ferric ion (Fe^3+^) into ferrous ion (Fe^2+^). Thus, 10 µL of the sample (BUO extract and the lysed cells sample diluted 1:5 in distilled water) was mixed with 140 µL of FRAP reagent. The FRAP reagent was prepared by mixing 1.3 mL of a 10 mM TPTZ (Sigma-Aldrich, Milan, Italy) solution in 40 mM HCl, 1.3 mL of 20 mM FeCl_3_ × 6H_2_O and 13 mL of 0.3 M acetate buffer (pH 3.6). The microplate was incubated for 30 min at 37 °C and the absorbance was read at 595 nm. The results were calculated by a Trolox (Sigma-Aldrich, Milan, Italy) standard curve obtained using different concentrations (3–400 µM) (Figure S1). Absorbances were recorded on a Synergy™ HT-multimode microplate reader. Briefly, as regards the FRAP assay at the cellular level, HepG2 and Caco-2 cells (1.5 × 10^5^/well) were seeded on a 24-well plate. The next day, cells were treated with the BUO at 10, and 25 μg/mL for 24 h at 37 °C under a 5% CO_2_ atmosphere. After incubation, cells were treated with H_2_O_2_ (1.0 mM) or vehicle (H_2_O) for 60 min, then lysed using 40 μL urea 8 M Subsequently, these were diluted 1:5 in distilled water and the FRAP assay was performed as above described.

### 2.8. ORAC Assay

The ORAC assay is based on the scavenging of peroxyl radicals generated by the azo 2,2′-azobis(2-methylpropionamidine) dihydrochloride (AAPH, Sigma-Aldrich, Milan, Italy). Briefly, 25 µL of BUO extract (with a final concentration of 0.5, 1.0, 5.0, and 10.0 µg/mL) was added to 50 µL sodium fluorescein (2.934 mg/L) (Sigma-Aldrich, MO, USA) and incubated for 15 min at 37 °C. Then, 25 µL of AAPH (60.84 mM) were added and the decay of fluorescein was measured at its maximum emission of 528/20 nm every 5 min for 120 min using a Synergy™ HT-multimode microplate reader (Biotek Instruments, Winooski, VT, USA). The area under the curve (AUC) was calculated for each sample subtracting the AUC of the blank. The results were calculated using a Trolox calibration curve (2–50 µM) (Figure S1).

### 2.9. Fluorometric Intracellular ROS Assay

The ROS assay was performed by a standard method with some slight modifications [20] and more detailed information are reported in Supplementary Material. Briefly, HepG2 (3 × 10^4^) and Caco-2 (5 × 10^4^) cells were incubated with 5 μL of BUO (1, 10, and 25 µg/mL) for 1 h in the dark. To induce ROS formation, H_2_O_2_ (0.5 mM) for 30 min at 37 °C in the dark was used. Fluorescence signals (ex./em. 490/525 nm) were recorded using a Synergy H1 microplate reader (Biotek Instruments, Winooski, VT, USA).

### 2.10. Lipid Peroxidation (MDA) Assay

The MDA assay was performed by a standard method with some slight modifications [20]. See Supplementary Material for detailed information. Briefly, HepG2 and Caco-2 (2.5 × 10^5^ cells/well) cells were treated with BUO extract (1, 10, and 25 µg/mL) for 24 h. The day after, cells were incubated with H_2_O_2_ (1 mM) or vehicle (H_2_O) for 30 min, then collected and homogenized in 150 μL ice-cold MDA lysis buffer. The MDA-TBA adduct was analyzed by measuring the absorbance at 532 nm using the Synergy H1 fluorescent plate reader (Biotek Instruments, Winooski, VT, USA). To normalize the data, total proteins for each sample were quantified by the Bradford method. 

### 2.11. Nitric Oxide Level Evaluation on HepG2 and Caco-2 Cells, Respectively

HepG2 and Caco-2 cells (1.5 × 10^5^/well) were seeded on a 24-well plate. The next day, cells were treated with the EVOO extract at different concentrations (1, 10, and 25 μg/mL) for 24 h at 37 °C under a 5% CO_2_ atmosphere. After incubation, cells were treated with H_2_O_2_ (1.0 mM) or vehicle (H_2_O) for 60 min, then the cell culture media were collected and centrifuged at 13,000× *g* for 15 min to remove insoluble material. NO determination was carried out following the conditions reported in the Supplementary Material.

### 2.12. iNOS Protein Level Evaluation by Western Blot Analysis

A total of 1.5 × 10^5^ HepG2 and Caco-2 cells/well were seeded on 24-well plates and incubated at 37 °C under a 5% CO_2_ atmosphere. The following day, cells were treated with 1, 10, and 25 μg/mL of the BUO extract in a complete growth medium for 24 h. Western Blot experiments were performed using primary antibodies anti-iNOS and anti-β-actin following conditions previously reported [21]. See Supplementary Material for further details. 

### 2.13. Caco-2 Cell Culture and Differentiation

For differentiation, Caco-2 cells were seeded on polycarbonate filters, 12 mm diameter, 0.4 µm pore diameter (Transwell, Corning Inc., Lowell, MA, USA) at a density of 3.5 × 10^5^ cells/cm^2^ in complete medium supplemented with 10% FBS in both apical (AP) and basolateral (BL) compartments for 2 d to allow the formation of a confluent cell monolayer. Starting from day three after seeding, cells were transferred to an FBS-free medium in both compartments, supplemented with ITS [final concentration 10 mg/L insulin (I), 5.5 mg/L transferrin (T), 6.7 μg/L sodium selenite (S); GIBCO-Invitrogen, San Giuliano Milanese, Italy] only in the BL compartment, and allowed to differentiate for 21 days with regular medium changes three times weekly [22].

### 2.14. Cell Monolayers Integrity Evaluation 

The transepithelial electrical resistance (TEER) of differentiated Caco-2 cells was measured at 37 °C using the voltmeter apparatus Millicell (Millipore Co., Burlington, MA, USA), immediately before and at the end of the absorption experiments. In addition, at the end of the absorption experiments, cells were incubated from the AP side with 1 mM phenol-red in PBS with CaCl_2_ and MgCl_2_ for 1 h at 37 °C, to monitor the paracellular permeability of the cell monolayer. The BL solutions were then collected and NaOH (70 µL, 0.1 N) was added before reading the absorbance at 560 nm in a microplate reader (Synergy H1, Biotek, Winooski, VT, USA). The phenol-red passage was quantified using a standard phenol-red curve. Only filters showing TEER values and phenol red passages similar to untreated control cells were taken into consideration for peptide transport analysis. 

### 2.15. Trans-Epithelial Transport of BUO Extract 

Prior to experiments, the cell monolayer integrity and differentiation were checked by TEER measurement as described above. Cells were then washed twice, and peptide absorption assayed. Absorption experiments were performed in transport buffer solution (137 mM NaCl, 5.36 mM KCl, 1.26 mM CaCl_2_, and 1.1 mM MgCl_2_, 5.5 mM glucose) according to previously described conditions [23]. The BUO extract absorption and the metabolism were assayed by loading the upper compartment with BUO extract (at the concentration of 100 and 200 μg/mL) in the AP transport solution (500 µL) and the lower compartment with the BL transport solution (700 µL). Transport experiments were conducted for 2 h. See Supplementary Materials for detailed information and conditions.

### 2.16. HPLC-DAD-MS Analysis for Evaluating the Trans-Epithelial Transport of BUO Extract

The dried cellular extracts were dissolved in 150 µL of EtOH:H_2_O 2:1 *v/v* and, after centrifugation at 16,900× *g* for 5 min, the supernatant was recovered and used for the analyses. The instrument was an HP 1260 MSD mass spectrometer with an API/electrospray interface (Agilent Technologies, Santa Clara, CA, USA). The column was a Poroshell 120, EC-C18 (150 mm × 3.0 mm id, 2.7 μm; Agilent, Santa Clara, CA, USA) with a precolumn of the same phase. The mobile phase was acetonitrile (A) and H_2_O at pH 3.2 by HCOOH (B). The following multistep linear gradient was applied: from 5% to 40% A in 40 min, to 88% A in 5 min, and then to 98% A in 10 min, with a final plateau of 3 min (total time 58 min); flow rate was 0.4 mL/min. For the MS detector, the conditions were: negative ion mode, gas temperature 350 °C, nitrogen flow rate 10.5 L/min, nebulizer pressure 35 psi (241 KPa), capillary voltage 3500 V, and fragmentation energy between 80 and 150 V. 

### 2.17. Statistical Analysis

Statistical analyses were carried out by One-way, and Two-way ANOVA followed by Tukey’s post-hoc analysis, respectively (Graph-pad Prism 8). Values were expressed as means ± SD; *p*-values < 0.05 were considered to be significant.

## 3. Results and Discussion

### 3.1. Antioxidant Activity of the BUO Extract

The in vitro antioxidant capacity of the BUO extract was tested at 0.5, 1, 2.5, and 5 µg/mL using the TAEC, FRAP, and ORAC assays, whereas the DPPH assay was tested in the range of 10–100 µg/mL. The phenolic composition of BUO, according to a previous study [15], is shown in Table S1.

**Figure 1:**
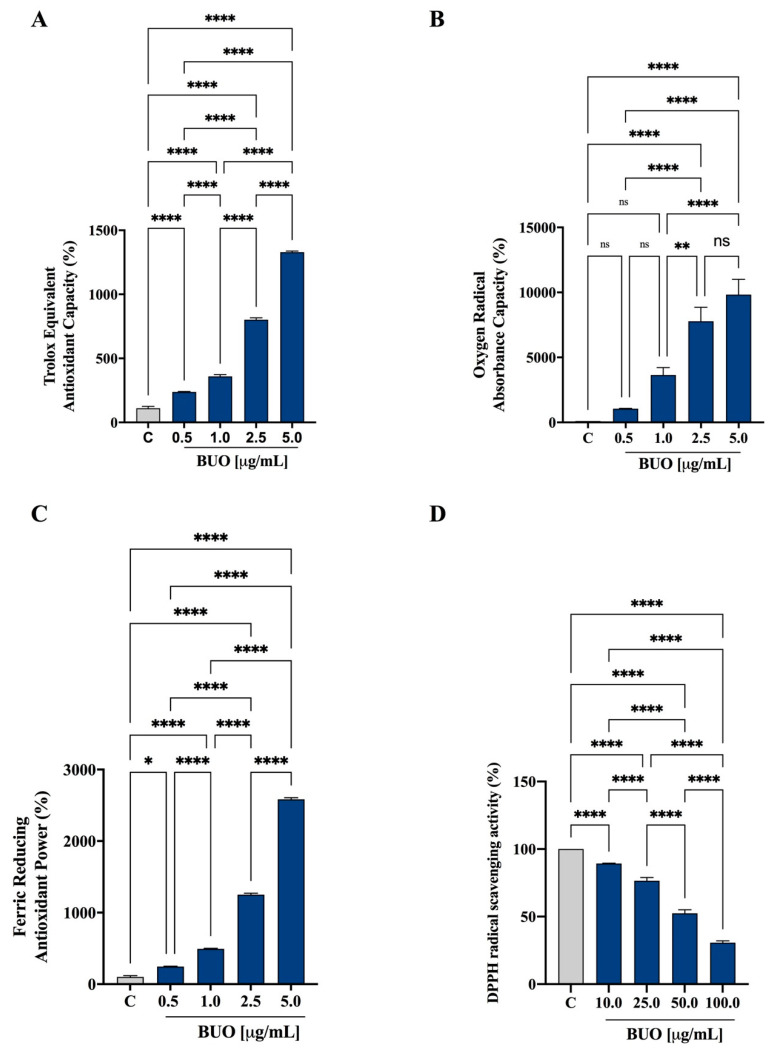
In vitro antioxidant power evaluation of the phenolic extract (BUO) extract by 2,2-azino-bis-(3-ethylbenzothiazoline-6-sulfonic acid (ABTS) (**A**), Oxygen Radical Absorbance Capacity (ORAC) (**B**), ferric reducing antioxidant power (FRAP) (**C**), and 2,2-diphenyl-1-picrylhydrazyl (DPPH) (**D**) assays. The data points represent the averages ± SD of four independent experiments performed in duplicate. All data sets were analyzed by One-way ANOVA followed by Tukey’s post-hoc test. ns: not significant and C: control sample (H_2_O). (*) *p* < 0.5; (**) *p* < 0.01; (****) *p* < 0.0001.

The BUO extract scavenged the ABTS radical by 214.9 ± 10.1%, 325 ± 41.1%, 728.2 ± 44.2%, and 1201.4 ± 25.6% at 0.5, 1, 2.5, and 5 µg/mL, respectively (*p* < 0.001, Figure 1A). In addition, in the ORAC test, this extract was able to scavenge the peroxyl radicals generated by 2,2’-azobis(2-methylpropionamidine) dihydrochloride up to 1059 ± 23%, 3638 ± 579%, 7782 ± 1070%, and 9837 ± 1175% versus the control sample at 0.5, 1, 2.5, and 5 µg/mL, respectively (*p* < 0.0001, Figure 1B). Figure 1C shows that the BUO extract increased the FRAP by 246.7 ± 12.8%, 493.2 ± 23.4%, 1250 ± 64.8%, and 2583 ± 73.7% at 0.5, 1, 2.5, and 5 µg/mL, respectively (*p* < 0.0001). Finally, as shown by Figure 1D, the same extract scavenged the DPPH radical by 10.7 ± 0.3%, 23.4 ± 2.3%, 47.6 ± 2.7, and 69.3 ± 1.4% at 10, 25, 50, and 100 µg/mL, respectively. In all the above assays, the response was dependent on the dose. 

All these results support the efficient antioxidant power of the BUO extract, which has a high content of Ole derivatives and a prevalence of HO-Tyr derivatives (445 µg/g) on Tyr derivatives (333 µg/g), which actively contribute to the in vitro scavenging activity. In a previous paper, the antioxidant activity of the phenolic EVOO extract, prepared with the same procedure from an Apulian EVOO from the cultivar Coratina, showed similar antioxidant activity, although this phytocomplex contained a prevalence of Tyr derivatives [17].

In parallel, the radical scavenging activity of the BUO extract was also evaluated at the cellular level in HepG2 cells and Caco-2 cells by using DPPH. Before conducting these experiments, however, it was necessary to perform MTT experiments to exclude any potential cytotoxic effect. As shown in Figure S2, after 48 h of incubation of Caco-2 cells at the highest concentration (200 µg/mL), the BUO extract slightly reduced cell viability by 7.5 ± 0.9%. This result is in line with a previous investigation showing that it significantly impairs the viability of HepG2 cells at 200 µg/mL, but not at lower concentrations [15]. 

The DPPH assay was performed directly on HepG2 and Caco-2 cell lysates, after treatment with the BUO extract at the fixed concentration of 25 µg/mL, which is roughly 10 times lower than the first cytotoxic dose on HepG2 cells. The BUO extract reduced the DPPH radical by 35.5 ± 8.3% and 22.8 ± 9.0% on HepG2 and Caco-2 cells, respectively (Figure S3). 

### 3.2. The BUO Extract Decreases the H_2_O_2_-Induced Oxidative Stress in Hepatic and Intestinal Cells

**Figure 2:**
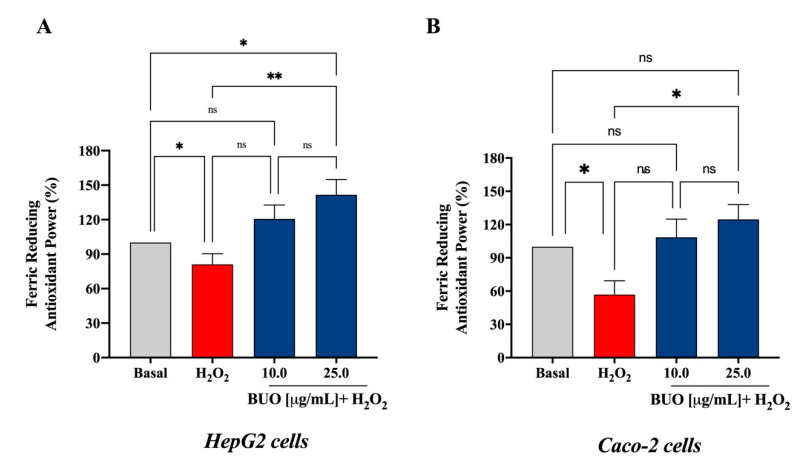
Antioxidant capacity of the BUO extract assayed at the cellular level, by ferric reducing antioxidant power (FRAP) assay. On (**A**) HepG2 and (**B**) Caco-2 cells, the BUO extract reverted the oxidative stress induced by 1 mM H_2_O_2_. Data represent the mean ± SD. of three independent experiments performed in duplicate. All data sets were analyzed by One-way ANOVA followed by Tukey’s post-hoc test. ns: not significant; (*) *p* < 0.5; (**) *p* < 0.01. Basal: untreated cells.

These preliminary results prompted us to carry out a deeper investigation on the antioxidant effect of the BUO extract at the cellular level, measuring its protective effect after induction of oxidative stress using H_2_O_2_ by the FRAP assay. As shown in Figure 2, treatment with 25 µg/mL of the BUO extract reverted the H_2_O_2_-induced oxidative stress in both HepG2 (Figure 2A) and Caco-2 (Figure 2B) cells. Furthermore, the experiments in HepG2 cells showed that 10 µg/mL of the BUO extract were sufficient to reverse the effects induced by hydrogen peroxide. More in detail, H_2_O_2_ (1 mM) decreased the antioxidant capacity measured by FRAP up to 81.0 ± 21.0% and 71.6 ± 27.8%, respectively, in HepG2 and Caco-2 cells. The pre-treatment of HepG2 cells with the BUO extract restored the FRAP up to 120.7 ± 31.7% and 141.6 ± 35.3% at 10 and 25 µg/mL, respectively, while the pre-treatment of Caco-2 cells increased FRAP up to 99.1 ± 33% and 124.6 ± 30%, respectively, at the same two concentrations (Figure 2A,B).

### 3.3. The BUO Extract Decreases the H_2_O_2_-Induced ROS in Hepatic and Intestinal Cells

**Figure 3:**
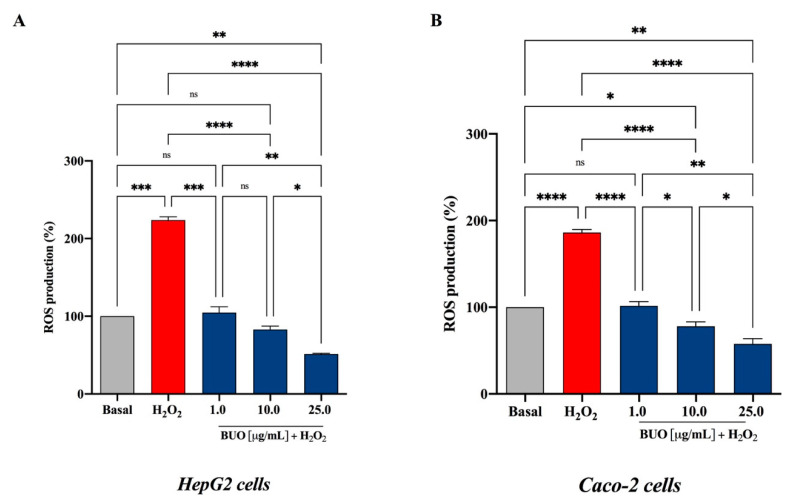
Evaluation of the effects of the BUO extracts on H_2_O_2_-induced reactive oxygen species (ROS) production levels in human hepatic HepG2 (**A**) and intestinal Caco-2 (**B**) cells. The data points represent the averages ± SD of six independent experiments in duplicate. Basal vs. H_2_O_2_ samples were analyzed by t-student test, whereas All data sets were analyzed by One-way ANOVA followed by Tukey’s post-hoc test. ns: not significant; (*) *p* < 0.5; (**) *p* < 0.01; (***) *p* < 0.001; (****) *p* < 0.0001. Basal: untreated cells.

The exposure of HepG2 and Caco-2 cells to H_2_O_2_ alone produced a dramatic increment of the intracellular ROS levels by 223.8 ± 4.3% and 186.2 ± 3.3%, respectively, versus the control cells (basal value = 100%, *p* < 0.5 in HepG2 cells and *p* < 0.001 in Caco-2 cells), whereas these increments were attenuated by the pre-treatment with the BUO extract in both cell lines (Figure 3A,B). In HepG2 cells, the BUO extract reduced the H_2_O_2_-induced intracellular ROS by 104.6 ± 7.6%, 82.9 ± 7.6%, and 51.4 ± 0.9%, respectively, at 1.0, 10, and 25 µg/mL (*p* < 0.0001) (Figure 3A), whereas in Caco-2 cells by 101.5 ± 4.9, 78.0 ± 5.1, and 57.6 ± 6.3 at 1, 10, and 25 µg/mL (*p* < 0.0001), respectively (Figure 3B). 

These findings suggest that after oxidative stress induced by H_2_O_2_, HepG2 cells are more susceptible than Caco-2 cells (*p* < 0.0001) to the ROS level production and that the pre-treatment with the BUO extract significantly protects both cell lines against the induced oxidative stress. Interestingly, despite the presence of any inducing stimulus, at the highest concentrations (10 and 25 µg/mL), the ROS levels are significantly reduced below the basal values in both HepG2 (*p* < 0.5) and Caco-2 cells (*p* < 0.01).

It is important to emphasize that, at 10 and 25 µg/mL, the BUO extract was much more effective in decreasing the H_2_O_2_-induced ROS levels in HepG2 cells (82.9 ± 7.6%, and 51.4 ± 0.9%, respectively) than the extract from the Coratina cultivar (198.8 ± 12.5% and 130.3 ± 11.3%, respectively) [17]. This difference may possibly be explained by their different phenolic profiles, since the BUO extract contains much more Ole derivatives (444.9 µg/g, measured as total OH-Tyr after hydrolysis), whereas the Coratina extract much more ligstroside derivatives (308.6 µg/g, measured as total Tyr after hydrolysis) [17]. In fact, OH-Tyr is a very efficient inhibitor of the ROS generation induced by tert-butylhydroperoxide (t-BOOH) in HepG2 cells [24].

### 3.4. The BUO Extract Decreases H_2_O_2_-Induced Lipid Peroxidation in HepG2 and Caco-2 Cells

**Figure 4:**
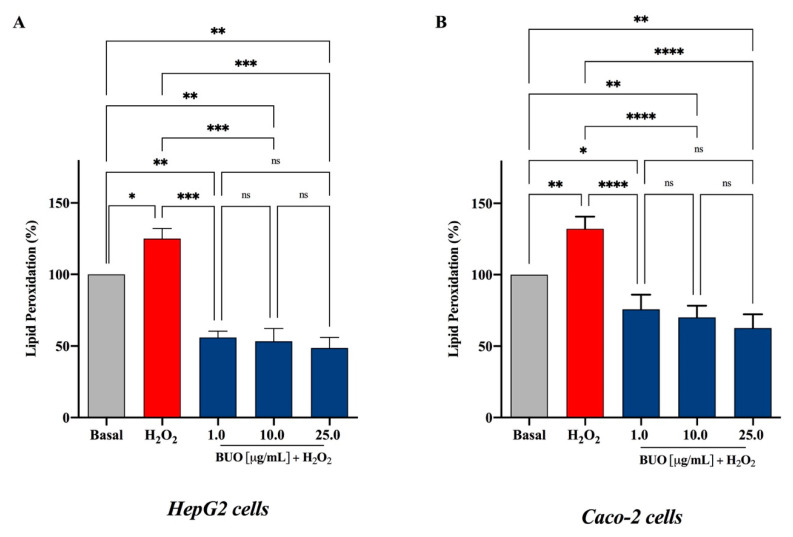
Evaluation of the effects of the BUO extract on H_2_O_2_-induced lipid peroxidation levels in human hepatic HepG2 cells (**A**) and intestinal Caco-2 cells (**B**) as assessed by intracellular MDA levels. The data points represent the averages ± SD of six independent experiments in duplicate. All data sets were analyzed by One-way ANOVA followed by Tukey’s post-hoc test. ns: not significant; (*) *p* < 0.5, (**) *p* < 0.01, (***) *p* < 0.001, (****) *p* < 0.0001. Basal: untreated cells.

The attack of oxygen free radicals on cellular lipids results in the formation of aldehydic lipid hydroperoxide decomposition products, such as MDA, which is traditionally considered a reliable marker of lipid peroxidation. Therefore, to link the BUO capacity of reducing the ROS production with its effect on the stability and integrity of hepatic and intestinal cells, lipid peroxidation was evaluated by intracellular levels of MDA after induction of oxidative stress using H_2_O_2_. The exposure of HepG2 and Caco-2 cells to H_2_O_2_ alone produced an increment of intracellular MDA levels by 125.1 ± 7.1% and 132.1 ± 8.5%, respectively, versus control cells (basal value = 100%, *p* < 0.0001 in HepG2 cells and *p* < 0.0001 in Caco-2 cells). This was attenuated by a pre-treatment with the BUO extract in both cell lines (Figure 4A,B). In fact, the BUO extract decreased the MDA levels below the basal ones in both cell models. In particular, in HepG2 cells (*p* < 0.001) the BUO extract diminished the H_2_O_2_-induced intracellular MDA by 55.9 ± 4.4%, 53.3 ± 8.9%, and 48.6 ± 7.3%, respectively, at 1, 10, and 25 µg/mL (Figure 4A), and in Caco-2 cells (*p* < 0.0001) by 75.8 ± 10.3, 70.1 ± 8.1, and 62.7 ± 9.6, respectively, at 1, 10, and 25 µg/mL (Figure 4B). After treatment with 1 mM H_2_O_2_, Caco-2 cells appeared to be slightly more sensitive to the MDA production than HepG2 cells, as shown by the increased MDA levels in HepG2 versus Caco-2 cells (*p* < 0.01). 

OH-Tyr and Tyr may be the most relevant phenolic compounds responsible for the modulation of intracellular lipid peroxidation levels. Indeed, clear evidence suggests that OH-Tyr protects the integrity of the HepG2 cellular membrane leading to a reduction of MDA levels after t-BOOH induced oxidative stress [24]. Similarly, Tyr reduces lipid peroxidation in HepG2 cells exposed to acute ethanol treatment [25]. Other evidence confirms that Tyr has a protective effect on membrane integrity also in other cellular systems. On the contrary, the Ole-mediated protective effect against oxidative stress is not associated with a reduction of MDA generation [26]. Interestingly, the BUO extract does not contain any intact Ole but only its non-glycosylated derivatives, which presumably show a greater ability to interact with membrane lipids.

### 3.5. The BUO Extract Modulates the H_2_O_2_-Induced NO Level Production via the iNOS Protein Modulation in HepG2 and Caco-2 Cells

ROS can act either as signaling molecules or as mediators of inflammation [27]. Superoxide can rapidly combine with NO to form reactive nitrogen species (RNS), such as peroxynitrite, with a reaction rate that is faster than the dismutation of superoxide by superoxide dismutase [28]. In addition, RNS leads to nitrosative stress, which parallels the pro-inflammatory activity of ROS [29]. Emerging evidence has clearly underlined the intricate relation between oxidative stress and inflammation [27].

**Figure 5:**
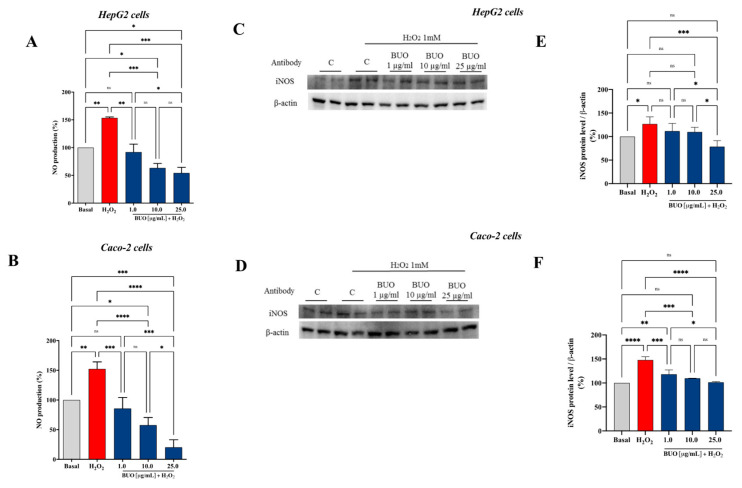
Effect of the BUO extract on the H_2_O_2_ (1 mM)-induced NO levels (**A**,**B**) and inducible nitric oxide synthase (iNOS) protein levels (**C**–**F**) in human hepatic HepG2 cells (A,C,E) and intestinal Caco-2 cells (B,D,F). The data points represent the averages ± SD of six independent experiments in duplicate. All data sets were analyzed by One-way ANOVA followed by Tukey’s post-hoc test. ns: not significant; (*) *p* < 0.5, (**) *p* < 0.01, (***) *p* < 0.001, (****) *p* < 0.0001. Basal: untreated cells.

Based on these considerations, the effects of the BUO extract on NO production were evaluated on both human hepatic HepG2 and intestinal Caco-2 cells, after oxidative stress induction. H_2_O_2_ (1 mM) treatment induced oxidative stress which led to an increase of intracellular NO levels up to 153.5 ± 18% and 152.4 ± 11.6%, respectively, in HepG2 and Caco-2 (Figure 5). Pre-treatment with the BUO extract reduced the H_2_O_2_-induced NO overproduction, reducing the values closer or even lower than the basal levels (*p* < 0.0001). Notably, the BUO extract reduced NO up to 92 ± 14.4%, 63.3 ± 8.1%, and 54.3 ± 10.2%, respectively, at 1, 10, and 25 µg/mL in HepG2 cells (*p* < 0.0001, Figure 5A), whereas, up to 85.7 ± 18%, 57.7 ± 12.8%, and 20.4 ± 12.6%, respectively, at 1.0, 10.0, and 25.0 µg/mL in Caco-2 cells (*p* < 0.0001, Figure 5B).

iNOS, is an enzyme expressed in different cell types [30] that is usually induced during inflammatory events [31]. The generation of NO by iNOS is associated with the alteration of NO homeostasis, which is linked to many pathophysiological conditions. In this study, the effect of the BUO extract on iNOS protein levels after oxidative stress induction was assessed by western blot experiments, in which the iNOS protein band at 130 kDa was detected and quantified (Figure 5C,F). After H_2_O_2_ treatment (1 mM), the iNOS protein increased up to 127 ± 14.8% (*p* < 0.5) and 148 ± 6.7% (*p* < 0.0001) in HepG2 and Caco-2 cells, respectively. In agreement with the modulation of NO production, the pre-treatment of both cell models with the BUO extract reduced the H_2_O_2_-induced iNOS protein, bringing their levels close to basal conditions. In particular, the BUO extract reduced iNOS levels up to 111.5 ± 16.5%, 109.8 ± 10%, and 78.6 ± 12.6% at 1, 10, and 25 µg/mL, respectively, in HepG2 cells (*p* < 0.01, Figure 5C,E), whereas in Caco-2 cells, they were reduced up to 118.3 ± 9%, 109.9 ± 0.2%, 101.4 ± 1.3% at 1, 10, and 25 µg/mL, respectively, (*p* < 0.001, Figure 5D,F).

Many studies underline the importance of EVOO phenols in limiting the NO production, but most of them are focused on the characterization of the effects of single phenols rather than of the total EVOO phytocomplex. In fact, it has been demonstrated that mostly OH-Tyr [32,33,34] can inhibit the NO overproduction induced by lipopolysaccharides (LPS) in monocytes and macrophages. In addition, glucuronide and sulfate metabolites of OH-Tyr and Tyr, together with their free forms, counteract the LPS-induced release of NO, acting as inhibitors of iNOS expression [35]. Only scarce evidence exists regarding the ability of complex EVOO extracts to impair NO overproduction. In agreement with our results, EVOO phenolic extracts from the Bosana cultivar (South of Sardinia) have been reported to limit oxysterols-mediated NO and cytokines overproduction, by modulating iNOS expression in Caco-2 cells [36]. These pieces of evidence together with our results highlight the need to direct more efforts to the characterization of the bioactivity of the whole EVOO phytocomplex for a fruitful valorization of this food, which is more complex than the sum of each single bioactive component [37].

### 3.6. Evaluation of the Steady-State Trans-Epithelial Transport of the BUO Extract Using Caco-2 Cells

**Figure 6:**
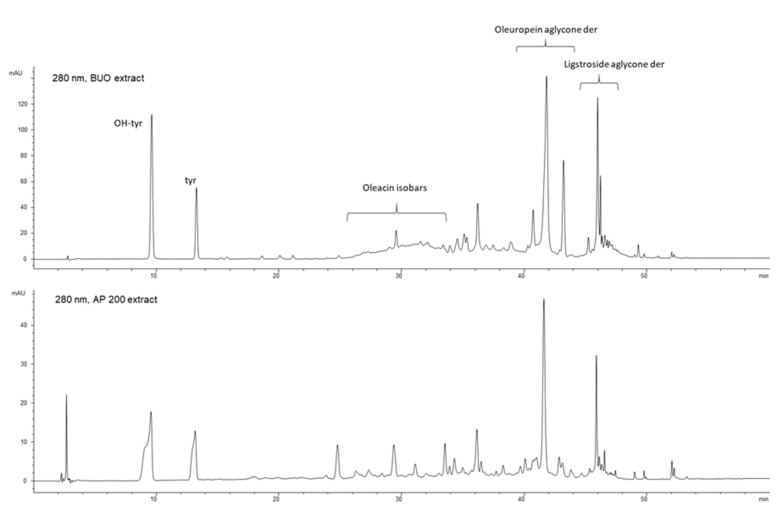
Chromatographic profiles at 280 nm showing OH-Tyr, Tyr, and the main secoiridoidic compounds in BUO and AP200 (AP side of the cells treated at 200 µg/mL) samples after 2 h incubation in the AP compartment.

**Figure 7:**
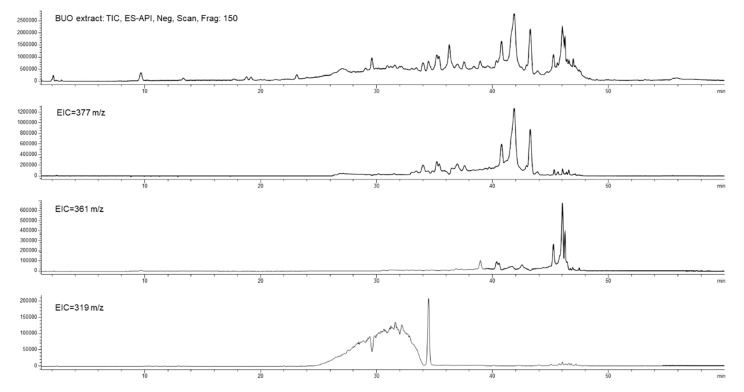

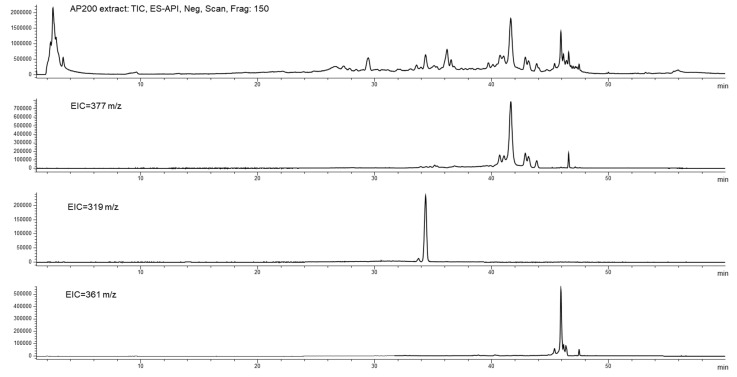
Total ion current (TIC) and extract ion current (IC) profiles of the BUO extract (**top**) and AP200 sample after incubation (**bottom**); in both the samples isobars of oleuropein aglycone at 377 *m/z*, isobars of oleacin at 319 *m/z*, and isobars of ligstroside aglycones at 361 *m/z*.

**Figure 8:**
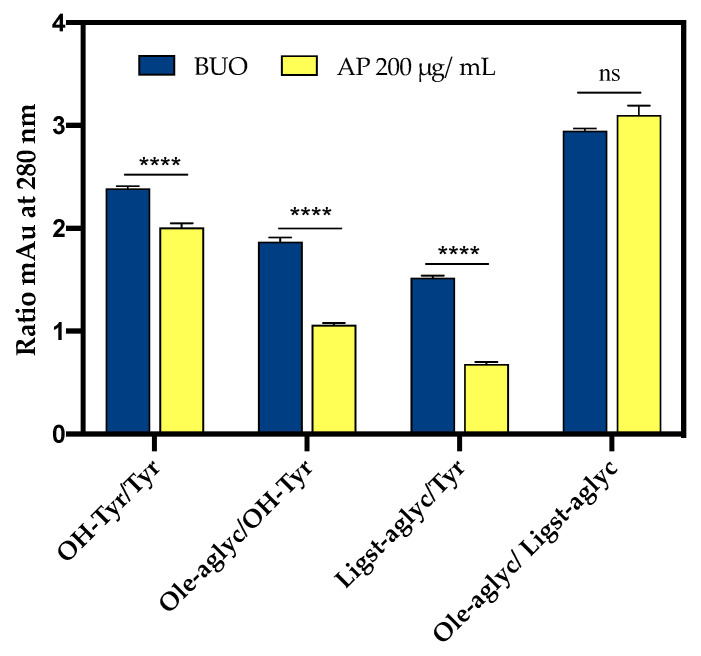
Specific ratios involving OH-Tyr, Tyr, Ole-aglyc, (rt 41.6 min) and ligstroside aglycone (Ligst-aglyc, rt 45.8 min), evaluated as area values at 280 nm for BUO extract and AP sample (AP 200 µg/mL) after 2 h incubation with 200 µg/mL BUO. (****, *p* < 0.0001). ns: not significant. Bars were analyzed by Two-Way ANOVA followed by Tukey’s test.

**Figure 9:**
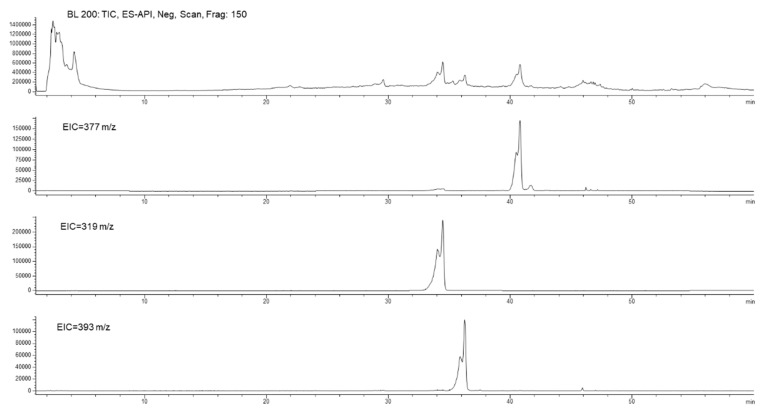
Chromatographic profiles of the BL sample after incubation for 2 h of 200 µg/mL BUO extract in the AP side of Caco-2 cells with, from top to bottom: TIC, EIC at 377 *m/z*, EIC at 319 *m/z*, and EIC at 393 *m/z*.

**Figure 10:**
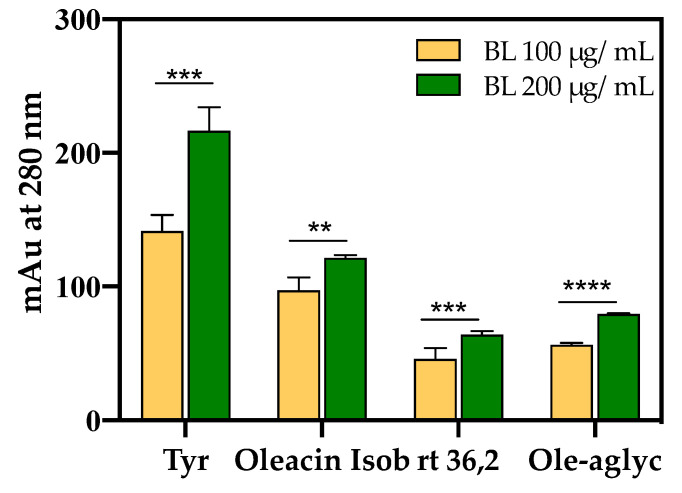
Main phenols in the BL samples were evaluated after incubation with the BUO extract at 100 and 200 µg/mL for 2 h, respectively. The data are the mean of triplicate experiments; the Ole aglycone isobar had rt 41.6 min, its oxidated derivative rt 36.2 and oleacin isobar rt 34.4 min (**, *p* < 0.01; ***, *p* < 0.001; ****, *p* < 0.0001). Bars were analyzed by Two-Way ANOVA followed by Tukey’s test.

The intestinal transport of polyphenols is potentially influenced by several affecting factors, such as the food matrix, biotransformation, and conjugation occurring during absorption [38,39]. Up to now, many studies have investigated the absorption of single EVOO phenols, demonstrating that both single OH-Tyr and Tyr are well transported but also metabolized by the intestinal cells [8]. On the contrary, little is known about the absorption of the total EVOO phytocomplex and how its complex composition may modulate the transport of single components. To fill this gap, the trans-epithelial transport of the BUO phytocomplex was investigated using differentiated Caco-2 cells. The steady-state study was designed to treat Caco-2 cells with the BUO extract at 100 and 200 µg/mL for 2 h. In all tested conditions, the treatment did not affect the monolayer integrity as monitored by TEER values and phenol red passage (data not shown). After 2 h of incubation, the AP and BL samples were collected from each filter, desalted, dried, and re-dissolved in a suitable solution to allow their analysis by HPLC-DAD-MS. The phenols recovered in the AP gave some information on the stability of the BUO extract components after incubation with the brush border of intestinal cells (Figure 6, Figure 7 and Figure 8). Instead, the phenols recovered in the BL side provided information on their transport by differentiated Caco-2 cells (Figure 9 and Figure 10).

#### 3.6.1. BUO Metabolism in AP Compartment

The chromatographic profiles of the AP solution of the untreated samples did not show any peak in the retention time range corresponding to the elution of the EVOO phenols (Supplementary Figure S4). Overall, the treated AP samples showed a profile very similar to that of the BUO extract (Figure 6), which has a phenolic composition summarized in Table S1. The small variation in peak shape of the most polar phenols (OH-Tyr and Tyr) was determined by the higher injected volume for the AP samples (10 µL), compared to the BUO extract (2 µL). 

With respect to BUO profile, changes involving the pool of secoiridoid derivatives [retention time (rt) range 28–34 min, rt 43 min, and rt 46 min], and the formation of some new minor compounds at 24.7 min, 26.2 min, and 27.3 min were observed in AP samples. It was hypothesized that these latter metabolites derive from the transformation of some phenols of the extract (Supplementary Figure S5). In particular, the metabolites at 26.2 and 27.3 min present a similar UV-Vis spectrum with a maximum absorption approximately at 360 nm and a shape different from those observed in the BUO extract. The metabolite at 24.8 min shows a UV-Vis spectrum more similar to the secoiridoid derivatives of oleuropein with a relative maximum at 280 nm, and a molecular ion [M-H]^−^ at 195 *m/z*. The available spectral data did not allow their identification so far.

Figure 7 shows the total ion current (TIC) and the extract ion (EI) profiles (EIC) for the BUO extract and the AP sample after 2 h incubation; the selected EIC profiles in Figure 7 were referred to as the isobaric forms of Ole aglycone at 377 *m/z*, of ligstroside aglycone at 361 *m/z* and of the decarboxylated form of Ole aglycone namely oleacin at 319 *m/z*. 

The UV-Vis spectra and the screening by mass spectrometry of the BUO extract confirmed the presence of several isobars of Ole aglycone and ligstroside aglycone, while the presence of oleocanthal (mw 304 Dalton) was not confirmed applying the EI at 303 *m/z*. After 2 h incubation, only one isobaric form of Ole aglycone (rt 41.6 min) and one of ligstroside aglycone (rt 45.8 min) were detected in the AP sample suggesting changes in the equilibrium among these species after interaction with the mature intestinal CaCo-2 cells. In relation to the isobaric forms of oleacin, another relevant difference between the BUO extract and the AP sample was observed: the EI profile of the BUO extract showed at 319 *m/z* a very wide unresolved peak which was not detected in the AP sample containing only one isobar of oleacin at 34.4 min. 

Conversely, the OH-Tyr and Tyr proportions, evaluated as the ratio of the areas at 280 nm, showed values of about 2.0 in the AP samples and about 2.3 in the BUO extract. This suggests a significant 13.1% reduction of the OH-Tyr/Tyr in the AP samples vs. the BUO extract (Figure 8). This difference may be explained considering that the interaction of both OH-Tyr and Tyr with intestinal cells may modulate their presence and stability. The comparison of other ratios of some main phenols detected in the BUO extract and AP sample (200 µg/mL) can help to summarize similarities and differences between these samples. Notably, a reduction of Ole-aglyc/OH-Tyr and Ligst-aglyc/Tyr ratios by 43.3% and 55.3%, respectively, in the AP samples (200 µg/mL) was observed in respect to the BUO extract. The lower values for Ole-aglyc/OH-Tyr and Ligst-aglyc/Tyr ratios in the AP samples may suggest that the little phenols OH-Tyr and Tyr are more transformed and absorbed than their precursors by Caco-2 cells. Further confirmation of this hypothesis is furnished by the same value obtained for Ole-aglyc/Ligst-aglyc in the BUO extract and the AP samples.

#### 3.6.2. BUO Phenols Transport to the BL Compartment

The chromatographic profiles of the BL samples after 2 h incubation of the BUO extract in the AP compartment were simpler than those of the AP samples. This suggests a selective transport of only some of the BUO phenols by Caco-2 cells into the BL compartment. Only Tyr, one isobaric form of the oleacin, and one of the Ole-aglyc were found, while OH-Tyr and the isobars of the aglycone ligstroside were not detectable (Figure 9). The identification of the isobar of oleacin was further confirmed by the presence in its mass spectrum of the ion corresponding to the dimer specie (639 *m/z*) together with the [M-H]^−^ molecular ion at 319 *m/z*. Notably, the metabolite at 36.2 min, according to the UV-Vis and MS spectra (Supplementary, Figure S6) was identified as an oxidation product of the Ole aglycone (mw 394 Dalton). This compound, detected in the BUO and AP sample, reached the BL compartment showing a concentration comparable to that of Ole aglycone.

Similarly, a recent study, investigating EVOO phenol extract absorption across Caco-2/TC7, has described five caffeic acid derivatives recovered from the BL side of the cells, which were not detected in the AP compartment [40]. This evidence contributes to confirming the active contribution of the intestinal epithelium in the modification of the phenolic profile of the EVOO phytocomplex. 

Figure 10 shows the four major phenolic compounds, namely Tyr, oleacin, rt 36.2, and Ole-aglycone, detected in the BL samples after 2 h incubation with the BUO extract (100 and 200 µg/mL) in the AP side of Caco-2 cells. More in detail, an increase in the amount of the compounds transported to the BL side was observed by doubling the BUO concentration in the AP compartment, although they did not double as expected by linear passive transport. This result may be explained considering that the phytocomplex might be transported with dynamics that differ from those of single phenolic entities tested alone.

In a previous paper, Manna and co-workers [41] showed that in Caco-2 cells OH-Tyr transport occurs via a passive bidirectional diffusion mechanism and that 3-hydroxy-4-methoxyphenyl ethanol is the main metabolite (approx 10% of the initial amount of OH-Tyr). Furthermore, the authors observed that the OH-Tyr absorption was not modified after incubation in the presence of structurally related phenols. Recently, D’Antuono et al. [40] performed a study aimed at characterizing the absorption of a phenolic extract obtained from Apulian naturally debittered table olives of the Bella di Cerignola cultivar (Italy), in which the debittering leads to the hydrolysis of Ole, the compound responsible for the characteristic bitter taste. Their results showed that OH-Tyr and Tyr, followed by verbascoside and luteolin, were among the best absorbed phenolic compounds by Caco-2 cells/TC7. In our study, however, the transepithelial transport of OH-Tyr was not observed. Although after 2 h of incubation the AP samples showed a higher amount of OH-Tyr compared to Tyr and almost the same OH-Tyr/Tyr ratio than in BUO extract, only Tyr appeared to be transported to the BL compartment. As for the Ole derivatives, only two selected isobaric forms of Ole aglycone and oleacin were found in the BL compartment, suggesting a selective release of the cells and/or an involvement of the other secoiridoid compounds in intracellular metabolic transformations into undetectable metabolites under the applied conditions. Differences in the major phenolic compounds shown to be transported by Caco-2 cells may derive from the use in our study of a whole phenolic extract instead of the single OH-Tyr [41] or to the different initial product, i.e., the EVOO versus debittered table olives [40].

## 4. Conclusions

Using a multidisciplinary strategy, our study supports the bioactivity of an EVOO phenolic extract through the comprehensive characterization of its antioxidant power both in vitro and at the cellular level. Hence, this is the first study aimed at evaluating the behavior of a real complex pool of phenolic compounds of an EVOO, rather than of single molecules, such as OH-Tyr or Ole, on differentiated human intestinal Caco-2 cells. The protective effect against the oxidative stress induced by H_2_O_2_ was demonstrated in two different cellular models, i.e., Caco-2 and HepG2 cells. In addition, our results showed for the first time a selective transepithelial transport of some Ole derivatives by differentiated Caco-2 cells. Further investigations into the effects of extracts obtained from different monocultivar EVOOs would be highly desirable to understand the mechanisms underlying the interaction between the different phenols and the intestinal cells.

## Supplementary Materials

The following are available online at https://www.mdpi.com/2076-3921/10/1/118/s1, Detailed description of Material and Methods, Figure S1: Trolox’s calibration curves obtained using TAEC (A), FRAP (B), ORAC (C), and DPPH (D) assays; Figure S2. Caco-2 cell vitality after treatment with BUO phenol extracts, Figure S3. Antioxidant activity evaluation of BUO by DPPH assay on HepG2 and Caco-2 cells at 25 µg/mL, Figure S4. Chromatographic profiles at 280 nm and TIC of the two controls: for Apical (AP) and basolateral (BL) samples. Figure 5S: UV-Vis spectra of some minor unidentified compounds found in the AP sample after incubation of two hours with BUO extract; UV-Vis spectrum (A) and mass spectrum in negative mode (B) of the compound at 36.2 min detected in BUO sample, in Apical (AP) and Basal (BL) samples; Table S1: Phenolic content in BUO (EVOO and dried extract) before and after acid hydrolysis expressed on EVOO (in µg/g) and on dry extract (in µg/mg) basis.

## Abbreviation

ABTS: 2:2-Azino-bis-(3-ethylbenzothiazoline-6-sulfonic acid); AP, apical; AUC, area under the curve; BL, basolateral; COX-2, cyclooxygenase 2; DMEM, Dulbecco’s modified Eagle’s medium; DPPH, 2,2-diphenyl-1-picrylhydrazyl; EFSA, European Food Safety Authority; EI, extract ion; EVOO, extra virgin olive oil; FBS, fetal bovine serum; FRAP, Ferric reducing antioxidant power; HDL, high-density lipoprotein; HMGCoAR, 3-hydroxy-3-methylglutaryl-coenzyme A reductase, OH-Tyr, hydroxytyrosol; iNOS, inducible nitric oxide synthase; ITS, insulin, transferrin selenium; LDLR, low-density lipoprotein receptor; MDA, malondialdehyde; MTT, 3-(4,5-dimethylthiazol-2-yl)-2,5-diphenyltetrazolium bromide; NO, nitric oxide; Ole, oleuropein; ORAC, Oxygen Radical Absorbance Capacity; PBS, Phosphate buffered saline; PMSF, Phenylmethanesulfonyl fluoride; ROS, reactive oxygen species; RT, room temperature; SDS-PAGE, Sodium Dodecyl Sulfate-Polyacrylamide; TEAC, Trolox Equivalent Antioxidant Capacity; TEER, transepithelial electrical resistance; TIC, total ion current; EIC, extract ion current Tyr, tyrosol; OH-Tyr, hydroxytyrosol.
