# Supplementary material for: Extra Virgin Olive Oil Phenolic Extract on Human Hepatic HepG2 and Intestinal Caco-2 Cells: Assessment of the Antioxidant Activity and Intestinal Trans-Epithelial Transport
Source: Antioxidants (Basel). 2021 Jan 15;10(1):118. doi: 10.3390/antiox10010118 (PMC7829860; doi:10.3390/antiox10010118)
Supplement: Supplementary file 1 [file antioxidants-10-00118-s001.pdf]

antioxidants

Supplementary Material: Extra Virgin Olive Oil Phenolic Extract on human hepatic HepG2 and intestinal Caco-2 cells: assessment of the antioxidant activity and intestinal trans-epithelial transport

Martina Bartolomei[1†], Bollati Carlotta[1†], Maria Bellumori[2], Lorenzo Cecchi[2], Ivan Cruz-Chamorro[1,3], Guillermo Santos-Sánchez[1,3], Giulia Ranaldi[4], Simonetta Ferruzza[4], Yula Sambuy[4], Anna Arnoldi[1], Nadia Mulinacci[2] and Carmen Lammi[1*]

Materials and Methods

1. Materials and cell cultures

All chemicals and reagents were of analytical grade. DPPH, ROS and lipid peroxidation (MDA) assays were from Sigma-Aldrich (St. Louis, MO, USA). The HepG2 cell line was bought from ATCC (HB-8065, ATCC from LGC Standards, Milan, Italy), whereas Caco-2 cells were obtained from INSERM (Paris, France) and were cultured following the conditions previously described (Lammi et al., 2015). The iNOS primary antibody came from Cell Signaling Technology (Danvers, MA, USA).

2. Cell culture

HepG2 cell line and Caco-2 cells were cultured in DMEM high glucose with stable L-glutamine, supplemented with 10% FBS, 100 U/mL penicillin, 100 µg/mL streptomycin (complete growth medium) with incubation at 37 °C under 5% (HepG2) or 10% (Caco-2) CO2 atmosphere. Caco-2 cells were routinely sub-cultured at 50% density. HepG2 cells were used for no more than 20 passages after thawing, because the increase in number of passages may change the cell morphology and characteristics and impair assay results.

3. Production of the EVOO extract

An EVOO sample produced by Società Agricola Buonamici SrL (Fiesole, Florence, Italy) in the 2017 olive oil campaign from monocultivar olives of the typical Tuscan cultivar Frantoio was used for the study (BUO oil). The BUO extract was obtained following the procedures previously described (Lammi et al., 2020). Briefly, the extraction of total phenols and their chromatographic analyses were carried out respectively with MeOH:H2O 80:20 v/v, and using a column SphereClone ODS (2) column, 250×4.6 mm (5µm) (Phenomenex, CA, USA), respectively, applying the analytical conditions reported by the IOC method [IOC/T.20/Doc No. 29]. An acidic hydrolysis was applied to the obtained extract for determining total OH-tyrosol (OH-Tyr) and total tyrosol (Tyr); the hydrolyzed extracts were not used for the biological tests, but only for analytical purposes. A RP18-Gemini column, 150×3 mm (5 µm) (Phenomenex, CA, USA) was used for the analysis of this latter extract. The Tyr content was evaluated at 280 nm using the calibration curve of Tyr standard (purity grade 98%), while the OH-Tyr content was expressed using the same calibration curve and applying a corrective factor (mg OH-Tyr = mg Tyr × 0.65).

4 3-(4,5-dimethylthiazol-2-yl)-2,5-diphenyltetrazolium bromide (MTT) assay

A total of 3 × 10^4 HepG2 cells/well and 5 × 10^4 Caco-2 cells/well were seeded in 96-well plates and treated with 25, 50, 100 and 200 µg/mL of BUO EVOO extract, or vehicle (H_2O) in complete growth media for 48 h at 37°C under 5% CO_2 atmosphere. Subsequently, the treatment solvent was aspirated and 100 µL/well of filtered 3-(4,5-

dimethylthiazol-2-yl)-2,5-diphenyltetrazolium bromide (MTT) solution was added. After 2 h of incubation at 37 °C under 5% CO_2 atmosphere, 0.5 mg/mL solution was aspirated and 100 μL/well of the lysis buffer (8 mM HCl + 0.5% NP-40 in DMSO) added. After 5 min of slow shaking, the absorbance at 575 nm was read on a Synergy H1 fluorescence plate reader (Biotek, Bad Friedrichshall, Germany).

5.2,2-. Diphenyl-1-picrylhydrazyl (DPPH) assay

The DPPH assay to determine the antioxidant activity *in vitro* and *in situ* was performed by a standard method with some slight modifications. For the *in situ* experiment, 3×10^4 HepG2 and Caco-2 cells/well were seeded in a 96-well plate, overnight in growth medium and the following day they were treated with the BUO extract at a concentration of 25 μg/mL for 24 h at 37 °C under 5% CO_2 atmosphere. The day after, cells were collected and homogenized in 100 μL/well ice-cold lysis buffer and samples were centrifuged at 13,000 g for 10 min at 4 °C. The supernatants were recovered and transferred into a new ice-cold tube. The DPPH solution (12.5 μM in methanol, 45 μL) was added to 15 μL of lysate or BUO EVOO extract at different concentrations (10 – 50 μg/mL) in a 96-well half area plate. The reaction for scavenging DPPH radicals was performed in the dark at room temperature and the absorbance was measured at 520 nm after 30 min incubation.

6. Trolox equivalent antioxidant capacity (TEAC) assay

TEAC assay is based in the reduction of ABTS (2,2-azino-bis-(3-ethylbenzothiazoline-6-sulfonic acid) radical induced by antioxidants. The ABTS radical cation (ABTS+·) was prepared by mixing a 7mM ABTS solution (Sigma) with 2.45 mM potassium persulfate (1:1) and stored for 16 h at room temperature and in dark. To prepare the ABTS reagent, the ABTS+· was diluted in 5 mM phosphate buffer (pH 7.4) to obtain a stable absorbance of 0.700 (±0.02) at 730 nm. For the assay, 10 μL of BUO extract (at final concentration of 0.5, 1, 5 and 10 μg/mL) were added to 140 μL of diluted the ABTS+·. The microplate was incubated for 30 min at 30 °C and the absorbance was read at 730 nm using a Synergy™ HT-multimode microplate reader. The TEAC values were calculated using a Trolox (Sigma) calibration curve (60 - 320 μM).

7. Ferric reducing antioxidant power (FRAP) assay

FRAP assay evaluated the ability of the sample to reduce ferric ion ($Fe^{3+)}$ into ferrous ion (Fe^{2+}). Thus, 10 μL of the sample (the lysed cells sample was diluted 1:5 in distilled water) was mixed with 140 μL of FRAP reagent. The FRAP reagent was prepared mixing 1.3 mL of a 10 mM TPTZ (Sigma) solution in 40 mM HCl, 1.3 mL of 20 mM $FeCl_3$~~x6H20~~ and 13 mL of 0.3 M acetate buffer (pH 3.6). The microplate was incubated for 30 min at 37°C and the absorbance was read at 595 nm. The results were calculated by a Trolox (Sigma) standard curve obtained using different concentrations (3-400 μM). Absorbances were recorded on a Synergy™ HT-multimode microplate reader.

8. Oxygen radical absorbance capacity (ORAC) assay

ORAC assay is based on the scavenging of peroxyl radicals generating by the azo 2,2′-azobis(2-methylpropionamidine) dihydrochloride (AAPH, Sigma). Briefly, 25 μL of BUO extract (with a final concentration of 0.5, 1, 5 and 10 μg/mL) was added to 50 μL sodium fluorescein (2.934 mg/L) (Sigma) and incubated for 15 min at 37 °C. Then, 25 μL of AAPH (60.84 mM) were added and the decay of fluorescein was measured at its maximum emission of 528/20 nm every 5 min for 120 minutes using a Synergy™ HT-multimode microplate reader. The area under the curve (AUC) was calculated for each sample subtracting the AUC of the blank. The results were calculated using a Trolox calibration curve (2-38 μM).

9. Fluorometric intracellular ROS assay

For cells preparation, 3×10^4 HepG2 cells/well and 5×10^4 Caco-2 cells/well were seeded on a 96-well plate overnight in growth medium. The day after, the medium was removed, 50 µL/well of Master Reaction Mix were added and the cells were incubated at 5% CO_2 and 37 °C for 1 h in the dark. Then, cells were treated with 5 µL of BUO extract to reach the final concentrations of 1, 10, and 25 µg/mL and incubated at 37 °C for 1 h in the dark. To induce ROS formation, cells were treated with H_2O_2 at a final concentration of 0.5 mM for 30 min a 37 °C in the dark and fluorescence signals (ex./em. 490/525 nm) were recorded using a Synergy H1 microplate reader.

10. Lipid peroxidation (MDA) assay

HepG2 and Caco-2 cells (2.5 × 10^5 cells/well) were seeded in a 24 well plate and, the following day, they were treated with the BUO extract at 1, 10, and 25 µg/mL for 24 h at 37 °C under 5% CO_2 atmosphere. The day after, cells were incubated with 1 mM H_2O_2 or vehicle (H_20) for 30 min, then collected and homogenized in 150 µL ice-cold MDA lysis buffer containing 3 µL of BHT (100×). Samples were centrifuged at 13,000 g for 10 min, then they were filtered through a 0.2 µm filter to remove insoluble materials. To form the MDA-TBA adduct, 300 µL of the TBA solution were added into each vial containing 100 µL samples and incubated at 95 °C for 60 min, then cooled to RT for 10 min in an ice bath. For the analysis, each reaction mixture (100 µL) was pipetted into a 96 well plate and the absorbance was measured at 532 nm using the Synergy H1 fluorescent plate reader. To normalize the data, total proteins for each sample were quantified by the Bradford method.

11. Nitric oxide level evaluation on HepG2 and Caco-2 cells, respectively

HepG2 and Caco-2 cells (1.5 x 105/well) were seeded on a 24-well plate. The next day, cells were treated with the EVOO extract at different concentrations (1, 10, and 25µg/mL) for 24 h at 37 °C under 5% CO_2 atmosphere. After incubation, cells were treated with H_2O_2 1 mM or vehicle (H_2O) for 60 min, then the cell culture media were collected and centrifuged at 13,000 g for 15 min to remove insoluble material. For the experiments, the supernatants were transferred in a 96-well plate and the buffer solution was added to each well in a ratio 1:2 to bring samples to final volumes of 50 µL and 40 µL for background and samples detection, respectively. Then, 5 µL of nitrate reductase solution and 5 µL of the enzyme co-factors solution were added to the samples and the plate was incubated at 25 °C for 2 h. Afterward, 25 µL of Griess Reagent A were added to each well and, after 5 min, 25 µL of Griess Reagent B were added for 10 min. For the detection step, the absorbance at 540 nm was measured using a Synergy H1 microplate reader.

12. iNOS protein level evaluation by western blot analysis

A total of 1.5 × 10^5 HepG2 and Caco-2 cells/well were seeded on 24-well plates and incubated at 37 °C under 5% CO_2 atmosphere. The following day, cells were treated with 1, 10, and 25 µg/mL of the BUO extract in complete growth medium for 24 h. The day after, cells were incubated with 1 mM H_2O_2 or vehicle (H_2O) for 60 min and, after each treatment, cells were scraped in 40 µL ice-cold lysis buffer (RIPA buffer + inhibitor cocktail + 1:100 PMSF + 1:100 Na-orthovanadate + 1:1000 β-mercaptoethanol) and transferred in ice-cold microcentrifuge tubes. After centrifugation at 13,300 g for 15 min at 4 °C, the supernatants were recovered and transferred into new ice-cold tubes. Total proteins were quantified by the Bradford's method and 50 µg of total proteins loaded on a pre-cast 7.5% sodium dodecyl sulfate-polyacrylamide (SDS-PAGE) gel at 130 V for 45 min. Subsequently, the gel was pre-equilibrated in H_2O for 5 min at room temperature (RT) and transferred to a nitrocellulose membrane (Mini nitrocellulose Transfer Packs,) using a Trans-Blot Turbo at 1.3 A, 25 V for 7 min. Target proteins, on milk or BSA blocked membrane, were detected by primary antibodies anti-iNOS and anti-β-actin. Secondary

antibodies conjugated with HRP and a chemiluminescent reagent were used to visualize target proteins and their signal was quantified using the Image Lab Software (Biorad, Hercules, CA). The internal control β-actin was used to normalize loading variations.

13. Caco-2 cell culture and differentiation

For differentiation, Caco-2 cells were seeded on polycarbonate filters, 12 mm diameter, 0.4 μm pore diameter (Transwell, Corning Inc., Lowell, MA, US) at a 3.5×10^5 cells/cm^2 density in complete medium supplemented with 10% FBS in both apical (AP) and basolateral (BL) compartments for 2 days in order to allow the formation of a confluent cell monolayer. Starting from day 3 after seeding, cells were transferred to a FBS-free medium in both compartments, supplemented with ITS [final concentration 10 mg/L insulin (I), 5.5 mg/L transferrin (T), 6.7 μg/L sodium selenite (S); GIBCO-Invitrogen, San Giuliano Milanese, Italy] only in the BL compartment, and allowed to differentiate for 18-21 days with regular medium changes three times weekly

14. Cell monolayers integrity evaluation

The transepithelial electrical resistance (TEER) of differentiated Caco-2 cells was measured at 37 °C using the voltmeter apparatus Millicell (Millipore Co., USA,), immediately before and at the end of the absorption experiments. In addition, at the end of the absorption experiments, cells were incubated from the AP side with 1 mM phenol-red in PBS with 1 mM $CaCl_2$ and 1mM $MgCl_2$ for 1 h at 37 °C, to monitor the paracellular permeability of the cell monolayer. The BL solutions were then collected and NaOH (70 μL, 0.1 N) was added before reading the absorbance at 560 nm by a microplate reader Synergy H1 from Biotek (Winooski, VT, USA). The phenol-red passage was quantified using a standard phenol-red curve. Only filters showing TEER values and phenol red passages similar to untreated control cells were considered for peptide transport analysis.

15. Trans-epithelial transport of BUO extract

Prior to experiments, the cell monolayer integrity and differentiation were checked by TEER measurement as described in detail above. Cells were then washed twice, and peptide absorption assayed. Absorption experiments were performed in transport buffer solution (137 mM NaCl, 5.36 mM KCl, 1.26 mM $CaCl_2$, and 1.1 mM $MgCl_2$, 5.5 mM glucose). In order to reproduce the pH conditions existing *in vivo* in the small intestinal mucosa, the AP solutions were maintained at pH 6.0 (buffered with 10 mM morpholinoethane sulfonic acid), and the BL solutions were maintained at pH 7.4 (buffered with 10 mM N-2-hydroxyethylpiperazine-N-4-butanesulfonic acid). Prior to absorption experiments, cells were washed twice with 500 μL PBS with 1mM $CaCl_2$ and 1mM $MgCl_2$ The BUO extract absorption was assayed by loading the AP compartment with BUO extract (100 and 200 μg/mL) in the AP transport solution (500 μL) and the BL compartment with the BL transport solution (700 μL). The plates were incubated at 37 °C and the BL solutions were collected 120 min. All BL solutions together with the AP solutions collected at the end of the transport experiment were stored at -80 °C prior to analysis. Three independent absorption experiments were performed, each in duplicate.

16. HPLC-DAD-MS analysis for evaluating the trans-epithelial transport of BUO extract

The dried cellular extracts were dissolved in 150 μL of the mixture EtOH:H_2O 2:1 v/v and, after centrifugation at 16,900 xg for 5 min, the supernatant was recovered and used for the analyses. The instrument was a HP 1260 MSD mass spectrometer with an API/electrospray interface (Agilent Technologies). The column was a Poroshell 120, EC-C18 (150 mm × 3.0 mm id, 2.7 *μ*m; Agilent, USA) with a precolumn of the same phase. The mobile phase was acetonitrile (A) and H_2O at pH 3.2 by HCOOH (B). The following multistep linear gradient was applied: from 5% to 40% A in 40 min, to 88% A in 5 min, and then to 98% A in 10 min, with a final plateau of 3 min (total time 58 min); flow rate

was 0.4 mL·min^{-1}. For the MS detector the conditions were: negative ion mode, gas temperature 350 °C, nitrogen flow rate 10.5 L/min, nebulizer pressure 35 psi (241 KPa), capillary voltage 3500 V and fragmentation energy between 80 and 150 V.

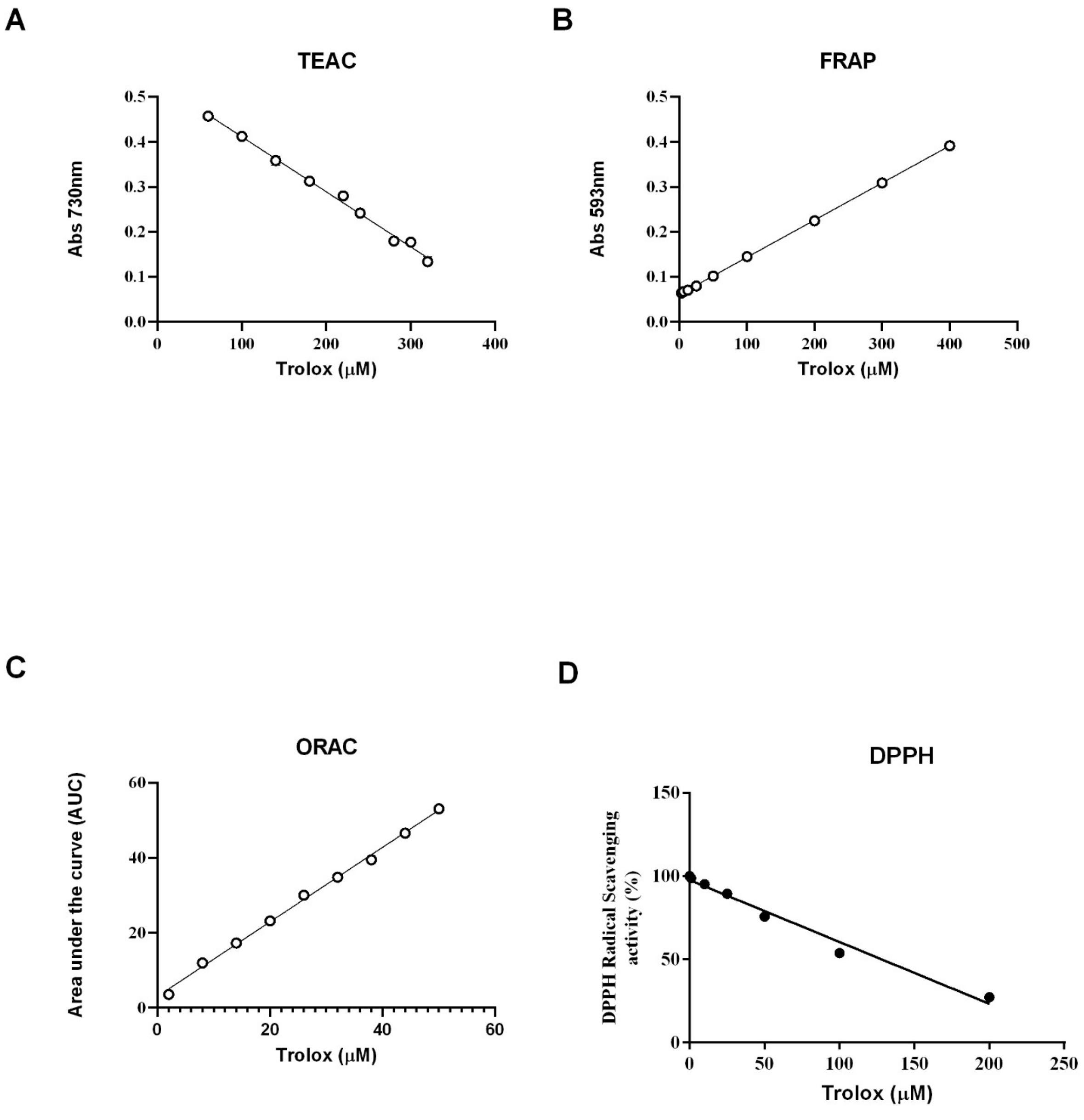

Figure S1: Trolox's calibration curves obtained using TAEC (A), FRAP (B), ORAC (C), and DPPH (D) assays.

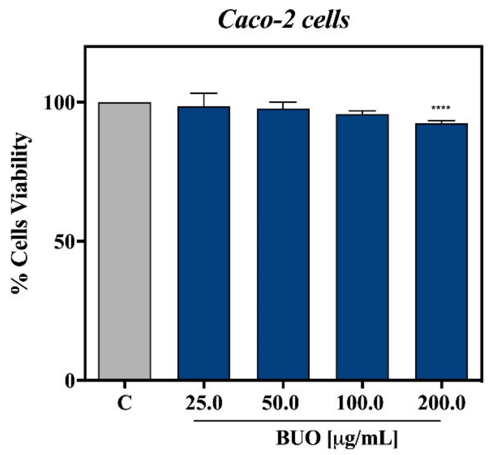

Figure S2. Caco-2 cell vitality after treatment with BUO phenol extracts by MTT assay. The BUO extract did not affect the Caco-2 vitality after 48 h of incubation up to 100 µg/mL, whereas at 200 µg/mL the cell viability was reduced by 7.5% (****) $p < 0.0001$. Data represent the mean ± SD of three independent experiments performed in triplicate. C: untreated Caco-2 cells.

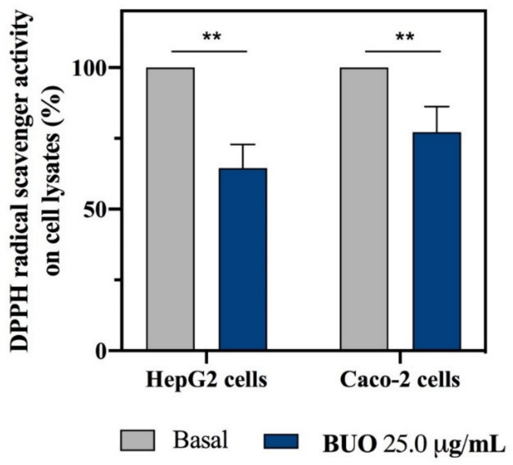

Figure S3. Cellular evaluation of the DPPH radical scavenger activity of BUO extract on HepG2 and Caco-2 cells lysates. The data points represent the averages ± SD of four independent experiments in duplicate. (**) $p < 0.01$.

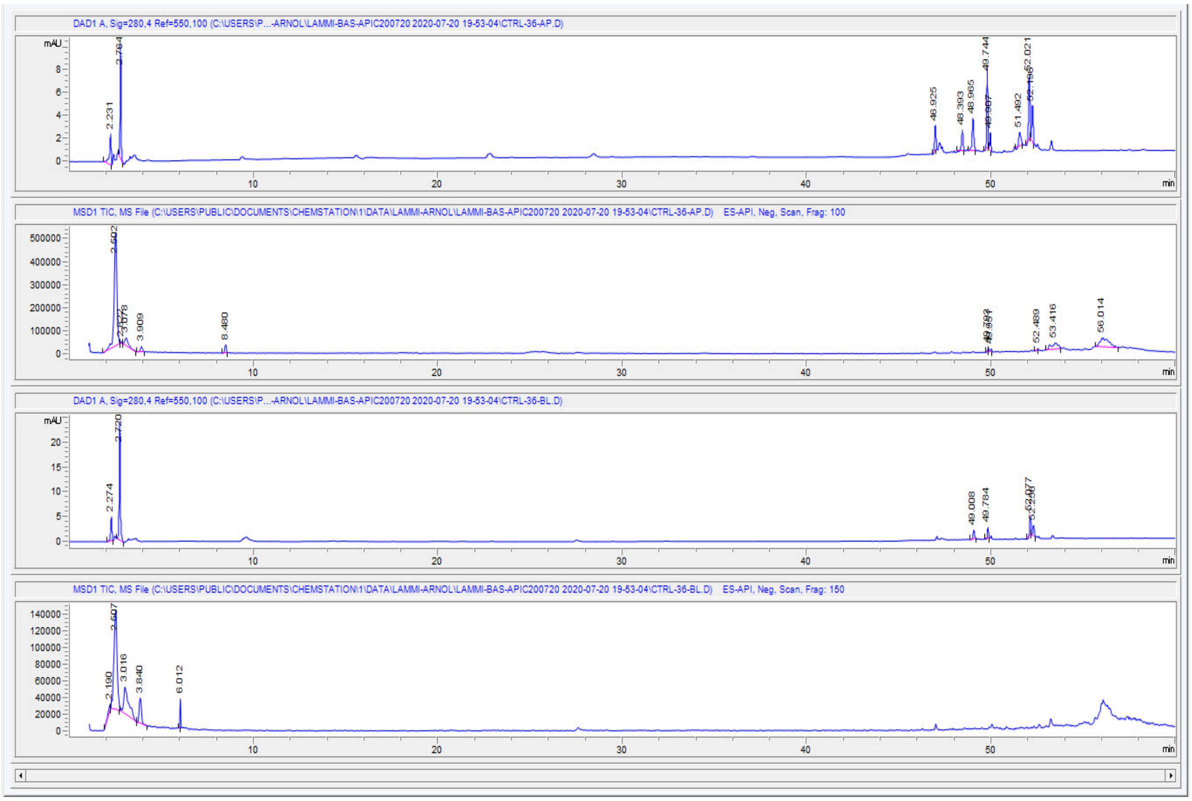

Figure S4. Chromatographic profiles at 280 nm and TIC of the two controls: Apical (AP) and basolateral (BL)samples

	**BUO** (before hydrolysis)	
	**EVOO (µg/g)**	**dry extract (µg/mg)**
Free hydroxytyrosol	9.3 ± 0.9	4.0 ± 0.1
Free tyrosol	5.1 ± 0.1	2.0 ± 0.1
Total Phenols	617.9 ± 34.1	289.3 ± 15.6
	**BUO** (after hydrolysis)	
	**EVOO (µg/g)**	**dry extract (µg/mg)**
Total hydroxytyrosol	444.9 ± 33.4	208.0 ± 15.6
Total tyrosol	332.9 ± 7.9	156.0 ± 3.9
Tyr+OH-tyr	777.8 ± 41.3	364.1 ± 19.5

Table S1 . Phenolic content in BUO (EVOO and dried extract) before and after acid hydrolysis expressed on EVOO (in µg/g) and on dry extract (in µg/mg) basis. Data are expressed as mean ± SD of three replicates. (According to Lammi, *et al.* . Extra Virgin Olive Oil Phenol Extracts Exert Hypocholesterolemic Effects through the Modulation of the LDLR Pathway: In Vitro and Cellular Mechanism of Action Elucidation. Nutrients: 2020; Vol. 12 (6), p 1723)

(A)

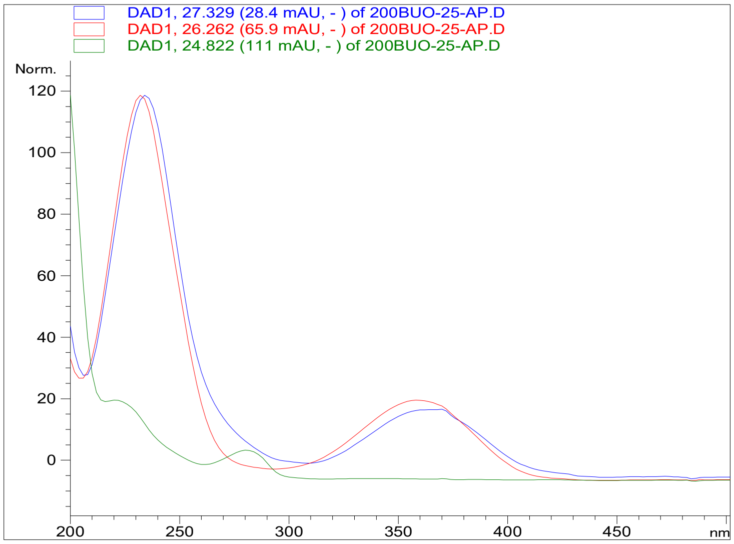

(B)

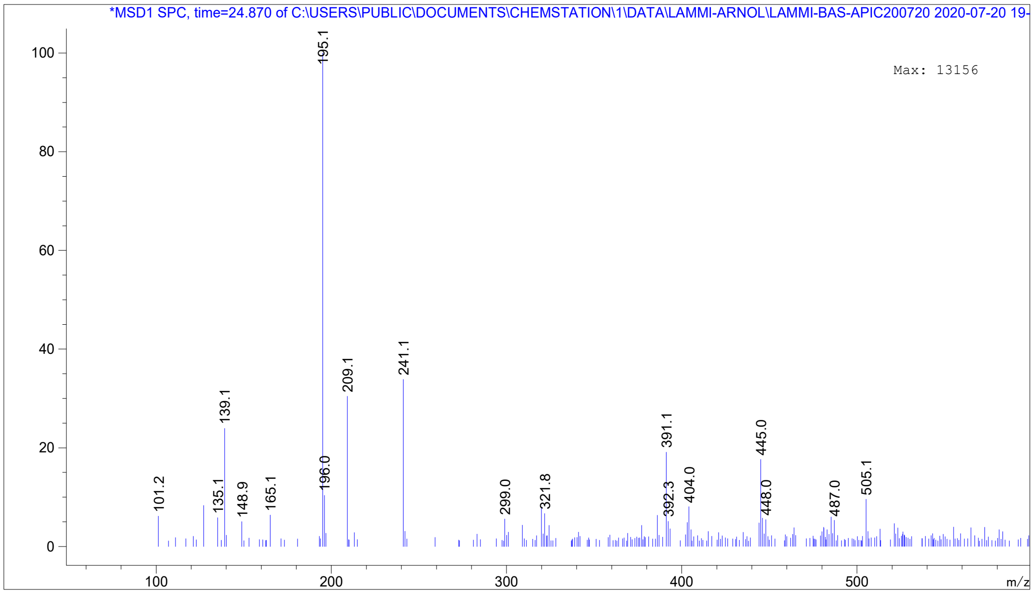

Figure S5. UV-Vis spectra of some minor unidentified compounds found in the AP sample after incubation of two hours with BUO extract: rt 24.7, 26.2 and 27.3 min (**A**); the mass spectrum of the compound eluted at 24.8 min in negative ionization mode (the only detectable within the three new metabolites) (**B**).

(A)

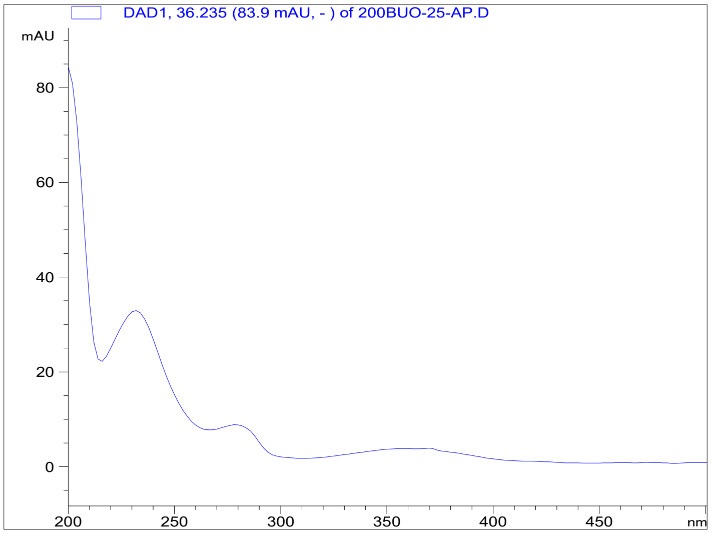

(B)

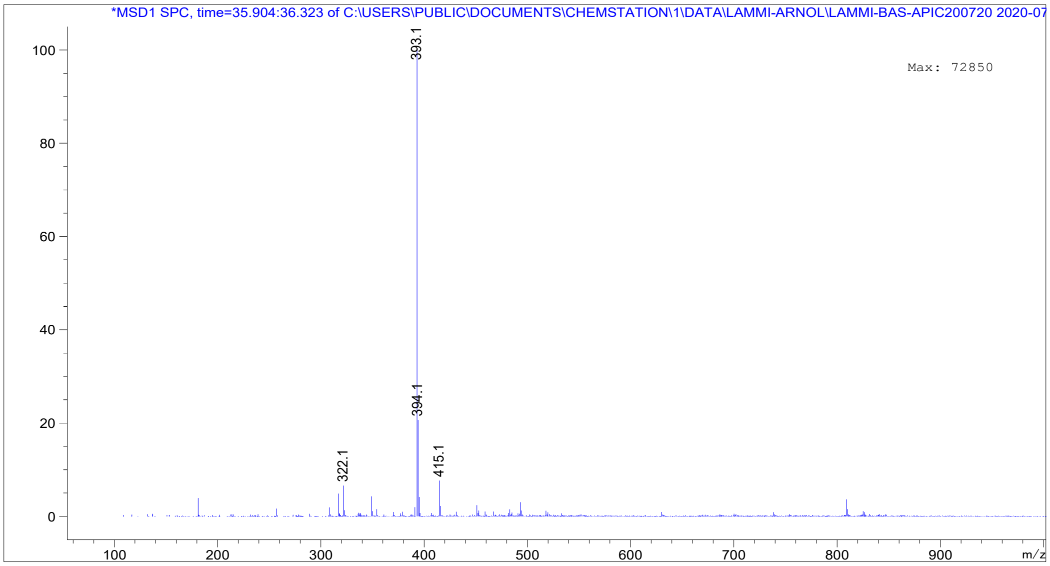

Figure S6. UV-Vis spectrum (**A**) and mass spectrum in negative mode (**B**) of the compound at 36.2 min detected in BUO sample, in Apical (AP) and Basal (BL) samples was tentatively identified as an oxidized derivative of oleuropein aglycone (mw 394 Dalton).
